# Hydrocarbon-Derived Prenetworked Carbon Nano-Onions for Wearable and Flexible Printed Microsupercapacitors

**DOI:** 10.1021/acsami.5c02847

**Published:** 2025-05-27

**Authors:** Ramu Banavath, Yufan Zhang, Sayyam Deshpande, Smita Shivraj Dasari, Stephnie Peat, Joseph V. Kosmoski, Evan C. Johnson, Micah J. Green

**Affiliations:** † Artie McFerrin Department of Chemical Engineering, Texas A&M University, College Station, Texas 77843, United States; ‡ Department of Material Science and Engineering, Texas A&M University, College Station, Texas 77843, United States; § Nabors Energy Transition Solutions LLC, Houston, Texas 77067, United States

**Keywords:** carbon nano-onions, carbon nanomaterials, microsupercapacitors, pyrolysis, morphology

## Abstract

Carbon nanomaterials have emerged as a promising solution for printed electronics, especially in microsupercapacitor (MSC) applications. This study examines the significance and compatibility of a newly developed industrial carbon nanomaterial derived from hydrocarbon streams via a scalable, catalyst-free process in a proprietary reactor. The carbon nanomaterials exhibit a unique morphology, characterized by nanoscale building blocks forming microscale networks, enhancing printed flexible electronics’ efficiency. Here, we utilize carbon nano-onions (CNOs) as an electrode material for MSCs. In addition to CNOs’ unique networked structure, high electrical conductivity, and large surface area make CNOs ideal for next-generation printed MSCs. The printed MSCs operate efficiently without metal current collectors, indicating that the printed electrodes with hydrocarbon-derived CNOs have sufficient conductivity comparable to that of metal-based current collectors. The printed MSCs demonstrated an excellent specific capacitance of 3.2 mF/cm^2^, outperforming many graphene-based MSCs. Additionally, these MSCs exhibited outstanding cycling stability, retaining 97% of their capacity after 10,000 galvanostatic charge–discharge cycles, and superior capacitance retention of 91% at a bending angle of 180°. These results indicate that the networked structure of CNOs maintains capacitance at various bending angles, confirming their high compatibility with flexible printed electronics. The integration of hydrocarbon-derived CNOs into printed electronics not only facilitates the development of lightweight, flexible, and cost-effective devices but also opens the door to innovative printed electronic applications.

## Graphical abstract

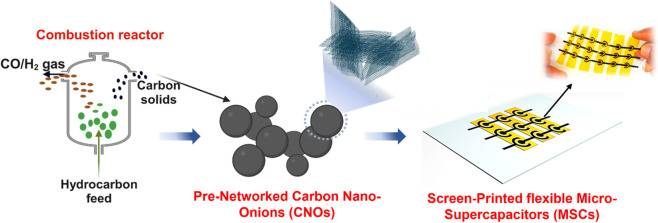

## 1 Introduction

Flexible microsupercapacitors (MSCs) are cutting-edge energy storage devices that merge high power density, rapid charge–discharge cycles, and mechanical flexibility, catering to the demands of modern electronic applications. Unlike traditional batteries, flexible MSCs are engineered to provide quick bursts of energy while withstanding mechanical deformation, making them ideal for use in flexible electronics, wearable devices, and portable sensors.^1^ These compact devices also offer remarkable cycling stability, which is essential for long-term operation in dynamic environments.^2^ A key factor in their efficiency and adaptability lies in the choice of electrode materials, which directly influences performance metrics such as energy density, cycle life, flexibility, and integration potential.

The primary function of flexible MSC electrodes is to store and release energy through either the electric double-layer capacitance (EDLC) or pseudocapacitance mechanism.^3^ Electrode materials for flexible MSCs must be highly conductive with a large surface area and, importantly, flexible enough to sustain performance under repeated bending, stretching, or compression cycles.^4^ Different materials explored for flexible MSC electrodes include conductive polymers (like polyaniline and poly­(3,4-ethylenedioxythiophene) (PEDOT))^5,6^ metal oxides (such as manganese dioxide, nickel oxide, and RuO_2_),^7–9^ transition metal carbides (MXenes),^10^ transition metal dichalcogenides (TMDs),^11,12^ metal–organic frameworks (MOFs),^13^ covalent organic frameworks (COFs),^14^ carbon-based materials (such as graphene and carbon nanotubes (CNTs)),^15^ and their hybrid composites.^16^

Each class of the aforementioned electrode materials for flexible MSCs provides unique benefits, but each also has specific drawbacks that may affect their practical application. For instance, conductive polymers such as polyaniline (PANI) and polypyrrole (PPy) are susceptible to oxidation in oxygen-rich environments, which causes them to degrade quickly and limits their cycle life.^17^ Metal oxides, such as manganese dioxide (MnO_2_) and ruthenium oxide (RuO_2_), deliver high capacitance, but their fragile nature limits their flexibility.^18^ While MXenes are conductive, they are susceptible to oxidation, and their synthesis processes are highly complex.^19^ TMDs offer good surface area and strong pseudocapacitive properties but have relatively low electrical conductivity.^20,21^ Additionally, their brittleness necessitates their hybridization with flexible materials. MOFs offer high surface areas but generally have poor conductivity, requiring conductive additives to perform effectively in MSCs.^22^ Moreover, MOFs can be chemically unstable in aqueous environments, which restricts their versatility.^23^ Carbon-based nanomaterials have been thoroughly investigated for MSCs among these different electrode materials due to their widespread availability, outstanding conductivity, high surface area, chemical stability, and intrinsic flexibility.^24^

Carbon nanomaterials, such as carbon black, activated carbon, carbon nanotubes, and graphene, exhibit unique morphologies and exceptional physical, chemical, and electrical properties. However, their effective utilization in flexible MSCs remains a significant challenge.^25^ Carbon black offers good conductivity, but forming a uniform flexible film is quite challenging.^26^ Activated carbon nanomaterials provide a large surface area for excellent capacitance, yet they lack sufficient conductivity and might require metals as current collectors.^27^ In contrast, carbon nanotubes (CNTs) and graphene-based nanomaterials are especially efficient for flexible printed MSCs because of their unique morphology, exceptional conductivity, and high specific surface area.^28–31^ These properties facilitate higher specific capacitance and help maintain performance under extreme mechanical stress. These nanomaterials are not widespread, largely because of concerns related to scalability of their synthesis process.^32^ For instance, the synthesis of CNTs relies on metal catalysts, which are difficult to remove.^33,34^ Graphene-based MSCs sometimes require conductive additives to allow for interlayer connectivity while maintaining high surface area.^35,36^ Therefore, developing a sustainable synthesis process and an effective electrode material with all the essential properties is important for practical flexible MSCs.

In this work, we have synthesized a unique morphology of prenetworked carbon nano-onion-type particles for flexible MSC electrodes. A new reactor method has recently been developed that allowed for the large-scale production of prenetworked carbon nano-onion nanoparticles from gaseous hydrocarbons.^37,38^ This reactor converts a hydrocarbon feedstock into carbon solids and CO/H_2_ gas instead of CO_2_, making the process carbon neutral. Unlike conventional combustion methods for synthesizing carbon nanomaterials, the feedstock used here is unique, though its specifics are proprietary.^39^ The resulting CO/H_2_ mixture is a valuable feedstock for producing various chemicals, including methanol, ethanol, olefins, and aromatics.^40–43^

In comparison to carbon nanotubes (CNTs) and graphene, these unique prenetworked structures of carbon nano-onion (CNO) particles can provide all the required electrode properties like conductivity, flexibility, and networked porous surface for flexible MSCs. In comparison with existing synthesis methods of carbon nanomaterials, the prenetworked CNOs can be produced in large quantities, making them more cost-effective and accessible. With the industrialization of this new carbon nanomaterial, evaluation of its properties and performance is essential for productization.

In this work, we have investigated the compatibility and performance of these prenetworked CNOs for flexible MSCs. The networked CNOs were used to prepare a screen-printable paste, which was then printed onto flexible polyethylene terephthalate (PET) sheets. The shear-thinning property of the CNO paste facilitated the printing of MSCs with the desired shape. With excellent electrical conductivity and a porous interconnected structure, the printed CNO-MSCs function without metal current collectors and demonstrate promise as an electrode material for printed flexible MSCs.

## 2 Experimental Methods and Characterization

### 2.1 Materials

Prenetworked CNOs (synthesized by Nabors Industries), ethyl cellulose (EC), terpineol, poly­(vinyl alcohol) (PVA), and phosphoric acid (H_3_PO_4_, 99%) were all purchased from Sigma-Aldrich. Polyethylene terephthalate sheets (1 mm thickness, purchased from McMaster-Carr).

### 2.2 Fabrication of Carbon Nano-Onions (CNO)-Based MSCs

The CNO-based paste was formulated by combining CNOs, ethyl cellulose (EC), and terpineol in a mass ratio of 3:1:10. This mixture was thoroughly ground in a mortar and pestle for 1 h to ensure a homogeneous paste suitable for screen printing. A stencil was designed according to the MSCs’ dimensions and applied to a prestretched aluminum screen printing frame, featuring 110-count/in. white monofilament polyester mesh fabric. Using a squeegee, the prepared CNO paste was spread onto the screen and manually printed onto PET sheets. The printed MSCs were then dried at 80 °C in a vacuum oven for 12 h. The effective loading of the active material after drying is 75%. Subsequently, an H_3_PO_4_/PVA gel electrolyte was prepared by adding 100 mg/mL PVA to a 20% H_3_PO_4_ solution, then heating the mixture in a water bath at 80 °C for 1 h to fully dissolve the PVA. After dissolution, the H_3_PO_4_/PVA gel electrolyte was placed under vacuum to remove bubbles formed during heating. A drop of the gel electrolyte was applied to the active area of the dried MSCs and the samples were cured overnight at room temperature. Finally, the cured MSCs were encapsulated with Kapton tape, making the encapsulated printed MSCs ready for performance evaluation.

### 2.3 Characterization Methods

#### 2.3.1 Scanning Electron Microscopy (SEM)

High-resolution field emission scanning electron microscopes (JEOL JSM-7500F and FEI QUANTA 600) were utilized to examine the morphologies of the CNOs and printed film. Before SEM measurements, each sample was sputter-coated with a 10 nm thick layer of Pt/Pd using a sputter coater (Cressington 208HR).

#### 2.3.2 Transmission Electron Microscopy (TEM) and High-Resolution Transmission Electron Microscopy (HRTEM)

TEM images and electron diffraction patterns were acquired using a Delong LVEM25, while high-resolution TEM (HRTEM) images were obtained with a Talos F200X G2 S/TEM. Each sample was prepared by dispersing in isopropyl alcohol (IPA) through sonication, followed by drop casting onto a lacey Formvar/carbon 200 mesh copper grid before TEM analysis.

#### 2.3.3 Raman Spectroscopy

Raman spectroscopy measurements were conducted using a confocal Raman microscope (JY Horiba with an Olympus BX 41 microscope) equipped with a 633 nm, 0.25 mW laser with a resolution of 0.16 cm^–1^.

#### 2.3.4 X-ray Powder Diffraction (XRD)

X-ray diffraction (XRD) characterization was performed using a Bruker D2 Phaser over a scanning range of 5–120°, with a step size of 0.02° and a scanning rate of 1.2°/min.

#### 2.3.5 X-ray Photoemission Spectroscopy (XPS)

XPS was measured on SPECS Enviro-ESCA with a mono X-ray source. All of the high resolution XPS spectra were measured with the same number of scans.

#### 2.3.6 Electrical Conductivity

The electrical conductivity of the CNO printed film was measured using the four-point probe method on a Resistivity Stand (S-302) obtained from Signatone Corporation, alongside a Keithley 2000 multimeter.

#### 2.3.7 Brunauer–Emmett–Teller (BET) Surface Area

N_2_ adsorption/desorption isotherms were measured by Anton Paar Nova 600 BET at 77 K to calculate the BET surface area. Before each measurement, the sample was heated to 300 °C and evacuated for 3 h.

#### 2.3.8 Rheological Properties

Rheological characterization of the CNO paste was performed on a stress-controlled rheometer (Anton Paar, MCR 301). The viscosity was evaluated as a function of shear rate (γ) in an interval of 0.01–100 s^–1^. The storage modulus (*G*′) and loss modulus (*G*″) were evaluated as a function of shear stress in an interval of 10^–1^ to 10^3^ Pa at an angular frequency of 1 rad/s. Three interval thixotropy was also performed on the ink at shear rates of 0.01, 100, and 0.01 s^–1^ to evaluate the viscosity recovery of the ink.

#### 2.3.9 Atomic Force Microscopy (AFM)

The topography and roughness of screen-printed films are analyzed using a Bruker Dimension Icon AFM with tapping mode.

#### 2.3.10 Electrochemical Characterization

All the electrochemical characterizations were carried out on a GAMRY potentiostat (GAMRY Reference 600). All cyclic voltammetry (CV) measurements were carried out with different scan rates (between 5 and 100 mV/s). All Electrochemical Impedance Spectroscopy (EIS) measurements were carried out in the frequency range of 0.01 Hz to 1 MHz with an amplitude of 10 mV. The galvanic charge–discharge (GCD) of the MSCs is carried out between 0 and 0.5 V. The active area of MSCs is 0.1256 cm^–2^. The areal capacitance, *C*_A_, is calculated from the GCD discharge curve according to the equation

1 $$C_{A}=I\cdot t_{d}/A\cdot \Delta E$$

where *I* is the applied current, *t*_d_ is the discharging time, *A* is the active area, and Δ*E* is the potential window during discharge experiments.

## 3 Results

Hydrocarbon-derived prenetworked CNOs were synthesized by Nabors Industries using their proprietary, reactor-based method. The specific light hydrocarbon used in this synthesis remains proprietary.^37,38^ In this novel process, different hydrocarbon gas inlet compositions are fed into the combustion reactor to generate carbon solids and CO/H_2_. By adjusting the gas composition, pressure, and temperature, a specific morphology of prenetworked CNO was synthesized; these CNOs are then utilized for the fabrication of MSCs. In contrast to traditional combustion methods used for carbon nanomaterial synthesis, our process is distinguished by its specific hydrocarbon feedstock composition and associated reactor conditions, designed to produce the material. Unlike CNTs or graphite-derived graphene, the carbon nanomaterials produced through this method are easier to synthesize in large quantities, making the process significantly more cost-effective and scalable. We have successfully and consistently achieved large-scale production yields ranging from 20 to 120 kg/day, highlighting the potential of this approach as a practical and industrially viable route for the hydrocarbon-based synthesis of high-quality carbon nanomaterials.

The structural hierarchy of the synthesized CNOs is illustrated in Figure 1. The CNO architecture comprises primary, secondary, tertiary, and quaternary structures. In the reactor, the combustion of hydrocarbons forms a primary structure of sp^2^-hybridized carbon atoms arranged into 6-membered hexagonal rings (can be confirmed from XRD and Raman spectra in Figure S2a,b). These primary repeats subsequently grow into two-dimensional (2D) graphene-like sheets to produce a secondary structure. Secondary sheets assemble upon one another through π stacking, eventually forming three-dimensional tertiary structures referred to here as nano onions. These spherical CNO particles aggregate due to electrostatic interactions, forming a microstructured quaternary network. SEM images in Figure 1b,c depict these prenetworked quaternary structures showing the aggregation of individual CNO particles, with sizes ranging from 80 to 100 nm. To gain additional insight into the nanoparticle structure, TEM imaging was performed. The low-resolution TEM image in Figure 1d further shows the prenetworked spherical particles. The HR-TEM imaging of CNOs was done on different particles to understand the structure. The HR-TEM image in Figures 1e and S1 reveals layered structures distributed throughout the particle, which confirms that the particles are highly crystalline in nature and resemble the similar HRTEM pattern of conventional CNOs. The prenetworked morphology of CNO particles is promising for the structural integrity of MSCs during strain.

**Figure 1:**
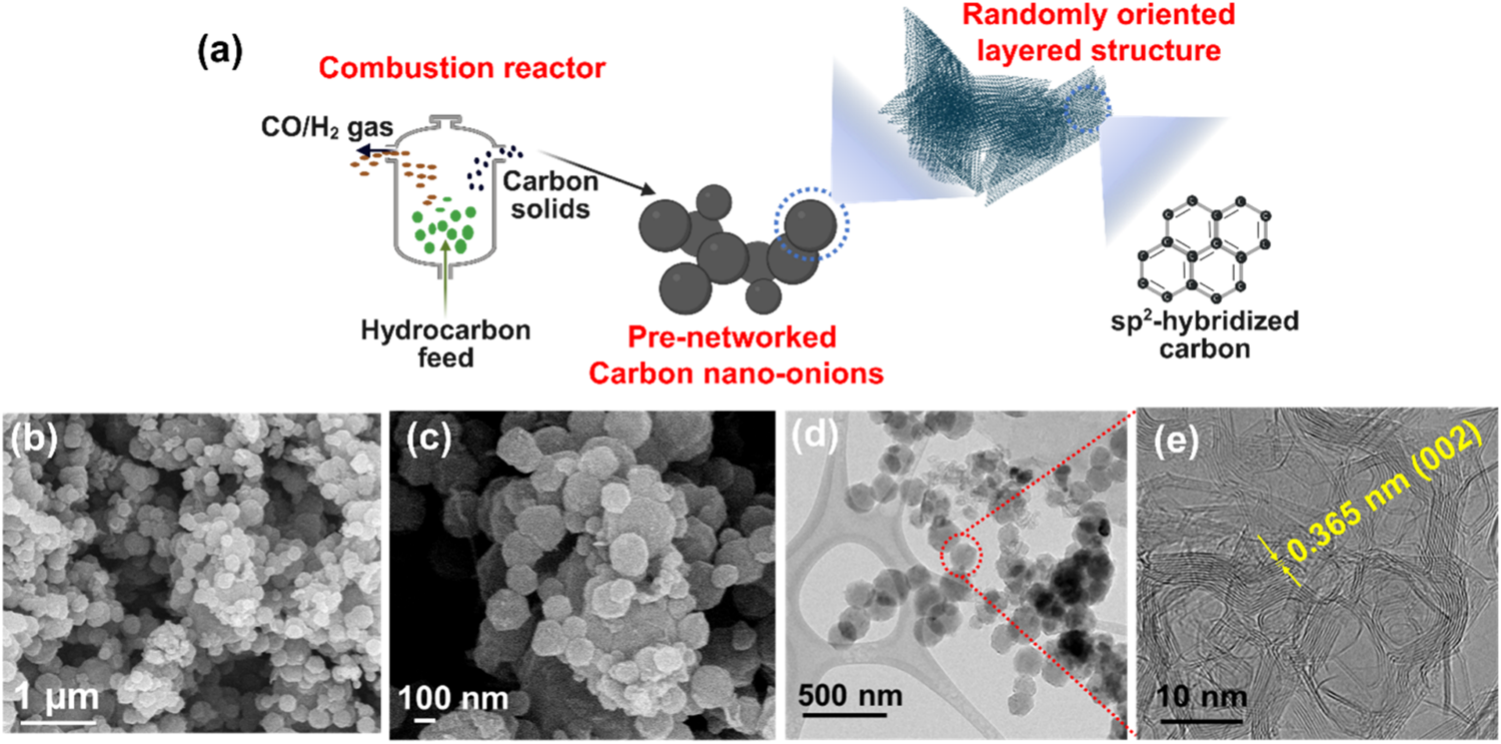
Structure of hydrocarbon-derived CNOs, (a) schematic CNO synthesis and structure, (b, c) low- and high-resolution field emission gun-SEM (FEG-SEM) images, (d) TEM image of CNOs, and (e) HR-TEM image of CNOs.

The XRD (Figure S2a) and Raman spectra (Figure S2b) of CNOs were analyzed to further understand the crystalline structure and quality (defect density) of the synthesized material. The XRD results reveal a (002) peak at a 2θ angle of 25.89°, which is shifted left compared to the (002) peak of bulk graphite at 26.54°. This leftward shift suggests an increased *d*-spacing between the graphitic layers in the CNOs, relative to graphite, which is quite common for CNOs.^44^ In the Raman spectra, a 2D peak appears around 2621 cm^–1^, also shifted left compared to graphite’s 2D peak at around 2700 cm^–1^, and the absence of a turbostratic hump in the 2D peak confirms the orderly graphene-like structure of the synthesized nanoparticles. An *I*_D_/*I*_G_ ratio of 0.22 suggests a low defect density, indicating high-quality CNOs as compared to traditional CNO-type nanomaterials.^45^

To further assess the chemical structure and purity, XPS analysis was conducted. The XPS survey spectrum of CNOs (Figure S2c) shows signals only for carbon and oxygen with no detectable impurities, indicating a high-purity carbon nanomaterial. Additionally, the deconvoluted high-resolution carbon XPS spectra (Figure S2d) show minimal carboxyl and carbonyl groups, further affirming the high quality and low defect density of the synthesized CNOs as compared to traditional CNOs.^45^ The synthesized CNOs have a BET specific surface area of 13 m^2^/g. The low specific surface area of CNOs arises from their multilayered structure, where nitrogen adsorption is limited to the outermost layers, while the inner layers remain inaccessible. The unique prenetworked morphology, structural characteristics, and purity of these CNOs suggest this is a promising candidate as an electrode material for flexible printed MSCs.

The synthesized CNOs from Nabors were then used to prepare a screen-printable paste for manufacturing printable MSCs, as schematically outlined in Figure 2. The paste was prepared using a mixture of CNOs, ethyl cellulose, and terpineol. This paste was printed on flexible PET sheets using MSC stencils created monofilament polyester mesh screen. The screen-printed MSCs were then dried and coated with an H_3_PO_4_/PVA gel electrolyte. After curing the gel electrolyte, the printed MSCs were encapsulated and used for further electrochemical testing.

**Figure 2:**
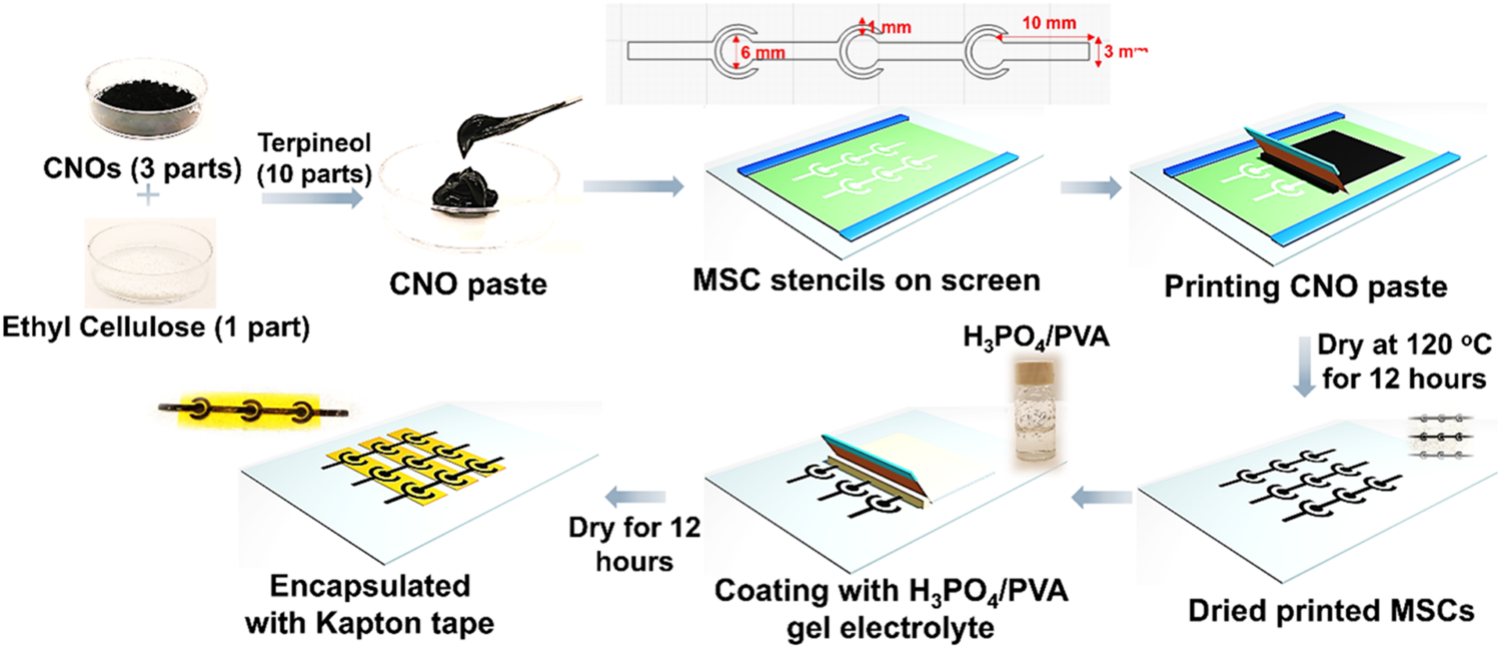
Schematic for the fabrication of CNO-based MSCs.

The CNO-based paste was synthesized by mixing CNOs, ethyl cellulose, and terpineol in a ratio of 3:1:10. A digital image of the CNO-based paste is shown in Figure 3a. Rheological measurements were carried out on the paste to understand its rheological properties. First, the viscosity of the paste was measured as a function of shear rate. The paste demonstrates shear thinning behavior as indicated by Figure 3b. Shear-thinning behavior is ideal for screen printing. Second, a three-interval thixotropy test was performed on the paste to evaluate the viscosity recovery of the paste after subjecting it to high shear. The plot shown in Figure 3c indicates that approximately 73% of the initial viscosity of the paste is retained after subjecting it to a shear rate of 100 s^–1^. Finally, the storage and loss modulus of the paste was evaluated as a function of shear stress, as shown in Figure 3d. Within the interval of 10^–1^ and 10^3^ Pa, the loss modulus (*G*″) is always greater than the storage modulus (*G*′), indicating that the viscous behavior dominates the elastic behavior. This behavior is commonly observed in systems with ethyl cellulose as a binder.^46,47^

**Figure 3:**
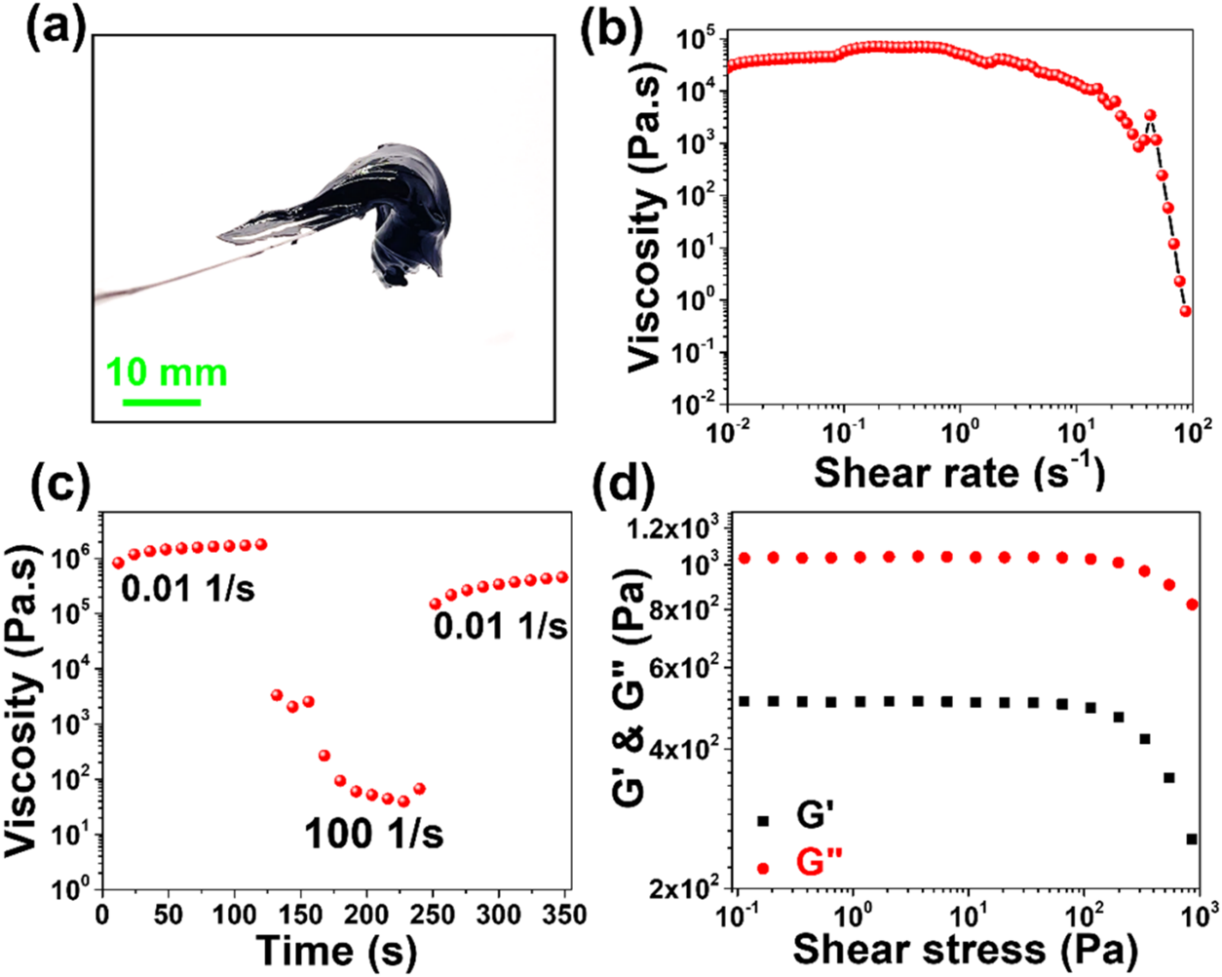
(a) CNO-based paste, (b) shear rate vs viscosity plot of CNO-based paste, (c) viscosity evolution of the CNO paste at alternating low and high shear rates, and (d) *G*′ and *G*″ as a function of the shear stress for the CNO paste.

The image of the printed CNO-MSCs shown in Figure 4a shows continuous printed electrode lines with no pin holes. SEM imaging was performed to observe the morphology of the printed CNO-based microsupercapacitors (Figure 4b). The morphology of the microsupercapacitors reveals a porous and interconnected microstructure, as shown in the high-resolution SEM image Figure 4c. The excellent electrical conductivity of the printed CNO film (Table S1), combined with the porous interconnected structure of the CNOs, is desirable for printed flexible MSCs to achieve a high capacitance. A porous and interconnected microstructure allows for high surface area for ion storage.^48^ SEM imaging was also done on the cross-section of the microsupercapacitor as shown in Figure 4d. The cross-section of the microsupercapacitor shows a uniform, porous, interconnected microstructure having a thickness of approximately 30 μm, indicating that the electrolyte can easily diffuse and access active sites in the film for ion storage. AFM was used to evaluate the surface roughness of the printed microsupercapacitor surface. Surface roughness variations can affect the ion diffusivity, charge transfer resistance, and electrode–electrolyte interactions, which influence the overall capacitive behavior of microsupercapacitors. A lot of studies have proved that the high roughness surface of electrodes promotes the adsorption of ions and results in enhancing the capacitance of EDLC-based supercapacitors.^49,50^ A porous surface with a root-mean-square (RMS) roughness of about 128 nm in the CNO-printed film is regarded as having a high surface roughness (Figure 4e). A high roughness gradient along the surface indicated the enhanced capacitive performance of the microsupercapacitors. The Raman spectrum of the printed CNO film in Figure S3 shows no significant change in the G, D, and 2D bands, confirming no change in the quality of CNO in the composite (CNO and EC).

**Figure 4:**
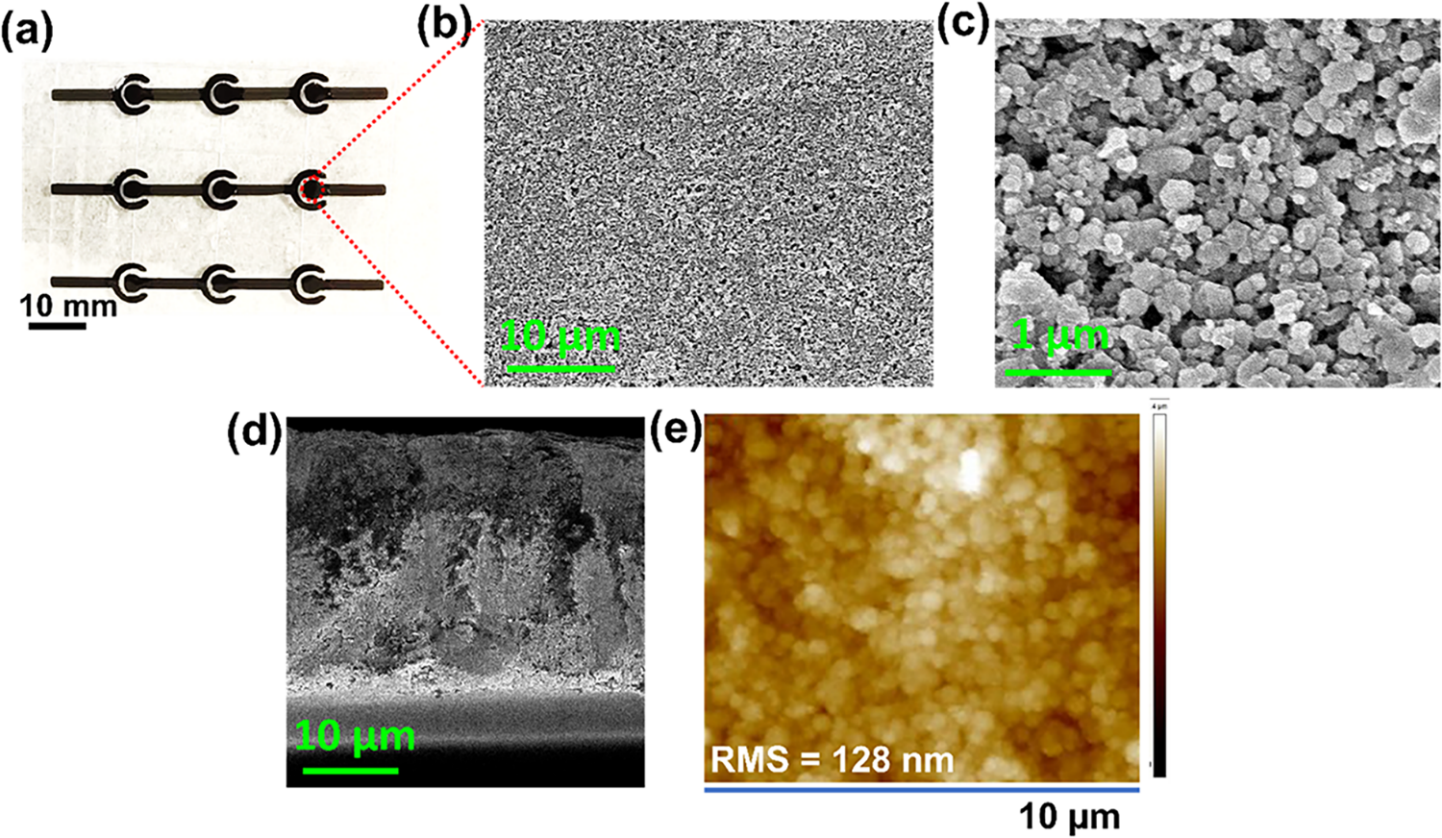
(a) Digital image of CNO-based printed MSCs after drying, (b) SEM of CNO-based printed film on PET substrate, (c) high-resolution SEM image, (d) cross-section of the printed film, and (e) AFM images of CNO-based printed MSC films.

### 3.1 Electrochemical Performance Evaluation of Printed CNO based MSCs

The cyclic voltammograms of the cells at different anodic limits, recorded at 50 mV/s, are shown in Figure 5a, while those at various scan rates are depicted in Figure 5b. As anticipated for a carbon-based material, the voltammograms exhibit an almost rectangular shape without redox peaks, confirming the material’s double-layer capacitive behavior. To better understand the charge storage mechanism in our system, we performed a *b*-value analysis based on the relationship between peak current (*i*) and scan rate (ν) from CV curves in Figure 5b, using eq 2.^51^

2 $$i=a\cdot v^{b}$$

**Figure 5:**
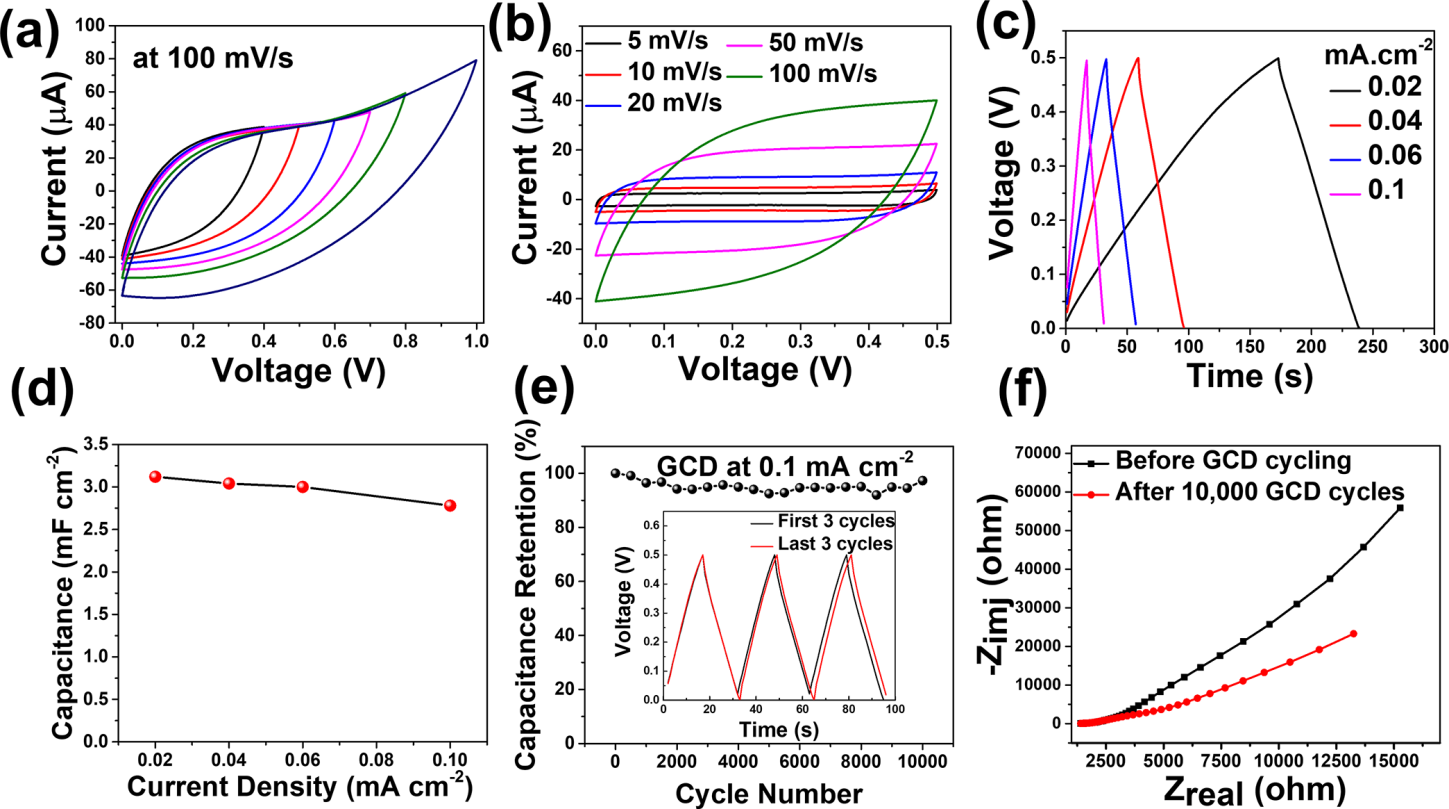
Electrochemical characterization of CNO-based printed MSCs of a single electrode, (a) CV in different voltage ranges at 100 mV/s, (b) CV at different scan rates, (c) GCD at different current densities, (d) current density vs specific capacitance plot from GCD plots, (e) cycling stability study at 0.1 mA/cm^2^ of current density, and (f) EIS before and after cycling of electrodes.

The calculated *b*-value around 0.76 (Figure S4) suggests that the charge storage is primarily capacitive in nature, with some contribution from diffusion-controlled processes. The GCD curves recorded at various current densities (20, 40, 60, and 100 μA/cm^2^) are presented in Figure 5c. These curves display an almost triangular shape, indicating minimal Ohmic drop and Coulombic efficiency of around 100%. The relatively low Coulombic efficiency observed in the GCD curves at high current densities may be attributed to side reactions, incomplete charge recovery, and kinetic limitations, including restricted ion diffusion within the electrode material.^52,53^ The cells exhibited an areal capacitance of 3.12 mF/cm^2^ at a current density of 20 μA/cm^2^ and maintained 2.78 mF/cm^2^ at a higher current density of 100 μA/cm^2^, retaining 89.1% of their initial capacitance (Figure 5d). This result indicates that the cells possess good rate capability. The CNO-based MSCs showed superior performance compared with previous studies using similar methodologies. For instance, earlier work with screen-printed graphene-based supercapacitors reported areal capacitances of 1.324 mF/cm^2^ at 12.5 μA/cm^2^ ^54^ and 1.0 mF/cm^2^ at 5 mV/s.^29^ Other fabrication methods, such as inkjet printing^55^ and flash foam stamp-inspired techniques,^56^ yielded areal capacitances of 0.7 and 4.02 mF/cm^2^, respectively, at 10 mV/s. Additionally, vertical graphene-based supercapacitors produced via chemical vapor deposition reached an areal capacitance of 1.06 mF/cm^2^ at a discharge current density of 0.1 mA/cm^2^.^57^ The detailed performance comparison with the existing literature of different carbon-based nanomaterials (Table S2) also shows comparable areal capacitance. A few reported graphene-based MSCs outperform the CNO-based MSCs, which may be attributed to the relatively low surface area of the CNOs. The fabricated MSCs exhibited excellent capacitance retention of 97.31% after 10,000 GCD cycles at the current density of 100 μA/cm^2^. The Coulombic efficiency of MSCs is approximately 100% even after 10,000 GCD cycles (Figure S5). The cycling stability of the device did not show any change in the resistivity of the device, as shown in the impedance spectra (Figure 5f). The high resistance observed in the EIS spectra likely originates from interfacial or contact resistances within the electrode structure or at the electrode–electrolyte interface, rather than from the intrinsic conductivity of the carbon nanoparticles themselves.^29,55,58^ This data collectively indicates that the prenetworked CNOs are promising for the practical use of printed MSCs. Postcycling morphology of the CNO-printed electrodes was examined using SEM imaging (Figure 6a,b). The electrodes exhibited no visible structural degradation or agglomeration of CNOs after 10,000 GCD cycles, indicating excellent mechanical and morphological stability. This also reflects the strong compatibility between the CNOs and the PVA/H_3_PO_4_ gel electrolyte throughout the prolonged electrochemical operation. To check the structural stability of CNOs after 10,000 GCD cycles, Raman spectroscopy was conducted (Figure S7a). The analysis revealed no notable change in the defect density, confirming the excellent structural stability of the CNOs. This observation aligns with the excellent capacitance retention and high Coulombic efficiency observed in CNO-based MSCs. A slight downshift in the 2D band position and increased broadening observed after cycling (Figure S7b) suggest a minor expansion in interlayer spacing. To evaluate the chemical stability of the CNO-printed electrode, XPS analysis was performed before and after 10,000 GCD cycles (Figure S8). The sustained high carbon content after cycling confirms the chemical integrity of the CNOs. However, a notable increase in the oxygen content was detected after cycling, which is likely due to the incorporation of oxygen from the PVA and H_3_PO_4_ components of the gel electrolyte.

**Figure 6:**
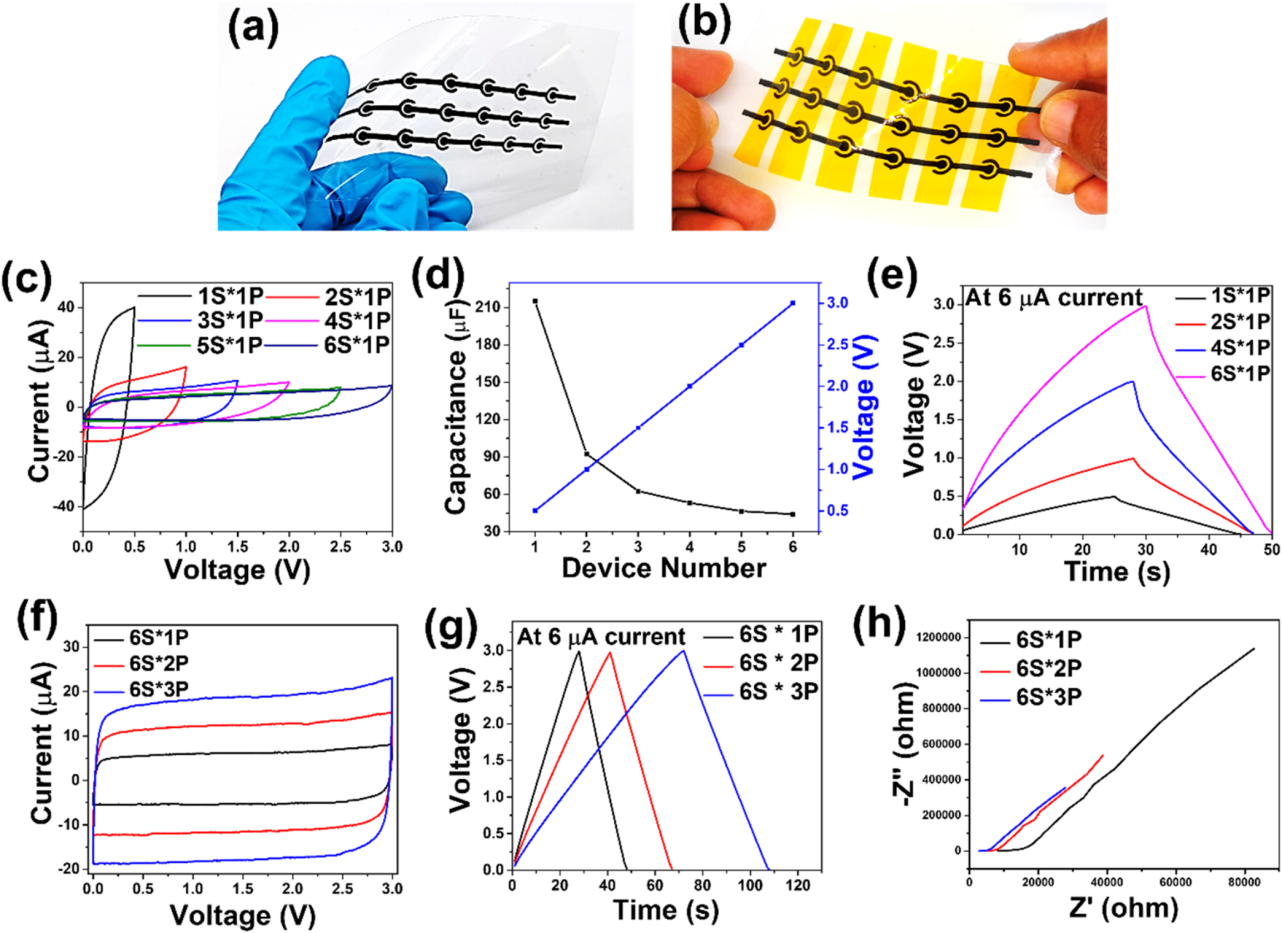
Electrochemical characterization of CNO-based printed integrated microsupercapacitors (IMSCs), (a, b) printed CNO-based MSCs connected in 6S × 3P before and after encapsulation in twisted state, (c) CV curves of MSCs connected in a tandem fashion of 1S × 1P, 2S × 1P, ···, 6S × 1P, obtained at 100 mV/s, (d) output voltage and capacitance as functions of serial cell number, calculated from Figure 5c, (e) GCD profiles of MSCs connected in a serial fashion of 1S × 1P, 2S × 1P, 4S × 1P, and 6S × 1P, measured at 6 μA, (f) CV curves of MSCs connected in 6S × 1P, 6S × 2P, and 6S × 3P obtained at 100 mV/s, (g) GCD profiles tested at 6 μA, and (h) EIS of MSCs connected in 6S × 1P, 6S × 2P, and 6S × 3P.

To address the needs of future microelectronics, the development of integrated power sources with flexible voltage and capacitance outputs is crucial. The shear-thinning behavior of the CNO paste, combined with excellent electrical conductivity and the strong electrochemical performance of prenetworked CNO electrodes, makes it an ideal material for printed MSCs. The excellent conductivity (Table S1) and outstanding electrochemical performance indicate that CNO-based printed electrodes are well-suited to function as both microelectrodes and current collectors. This enables the scalable production of integrated microsupercapacitors (IMSCs) with customizable voltage and capacitance outputs, tailored to meet specific application requirements. To demonstrate this feature, we fabricated a series of complex modular CNO-IMSCs (*x*S × *y*P), where *x* and *y* denote the number of cells connected in series and parallel, respectively. Figure 6a,b illustrates the printed CNO-IMSCs connected in a 6S × 3P configuration on the PET substrate, shown in the twisted state both before and after encapsulation. This demonstrates that the printed CNO electrodes exhibit robustness and remain intact on the PET substrate even under twisted conditions.

As anticipated, the CV curves for the CNO-IMSCs (*x*S × 1P, *x* = 1–6), connected in series from 1 to 6 cells and recorded at 100 mV/s, exhibited almost rectangular shapes, characteristic of typical EDLC behavior (Figure 6c). This configuration produced a stepwise linear increase in operating voltage from 0.5 to 3.0 V, while the current and capacitance gradually decreased (Figure 6c,d). This impressive series-capacitive behavior was also confirmed by the GCD profiles (Figure 6e). More importantly, the output capacitance can be readily enhanced using an in-parallel cell pack of CNO-IMSCs (6S × *y*P, *y* = 1–3). As shown in Figure 6f, the capacitance of CNO-IMSCs (6S × 1P) is doubled for configuration 6S × 2P and trebled for configuration 6S × 3P, while the operational voltage scan window was 0–3 V. The same observation can be seen in GCD plots of CNO-IMSCs (6S × *y*P, *y* = 1–3) shown in Figure 6g. The ideal tandem and parallel capacitive behaviors are further analyzed using EIS testing, where the equivalent series resistance (ESR) displayed an inverse relationship with the number of parallel rows in the cell pack (Figure 6h). This observation demonstrates the effectiveness of our approach for the cost-efficient mass production of integrated microscale energy storage packs, designed to provide customized performance that meets various real-world demands.

To evaluate the mechanical flexibility and stability of printed MSCs, CV measurements were conducted on 3S × 1P CNO-IMSCs under different bending conditions (as shown in Figure 7a). The CV curves across these bending states displayed minimal changes in the area and shape, as shown in the inset of Figure 7b, with the devices retaining approximately 91% capacitance at a 180° bending angle. Additional CV testing under repeated bending cycles (Figure 7c) demonstrated stable performance, with no significant changes in CV shape or area even after 1000 cycles, retaining 99.8% of their capacitance. These results highlight the excellent mechanical stability and flexibility of MSCs under mechanical stress and deformation. The impressive flexibility and mechanical stability of these devices can be attributed to the unique prenetworked structure of CNOs, which ensures consistent particle contact and conductivity across different bending states and bending cycles. The exceptional flexibility of CNO electrodes, coupled with the robust integrity of the microelectrodes and the incorporation of a gel electrolyte, presents significant potential for the seamless integration of CNO-based IMSCs into flexible microelectronics as a reliable power supply.

**Figure 7:**
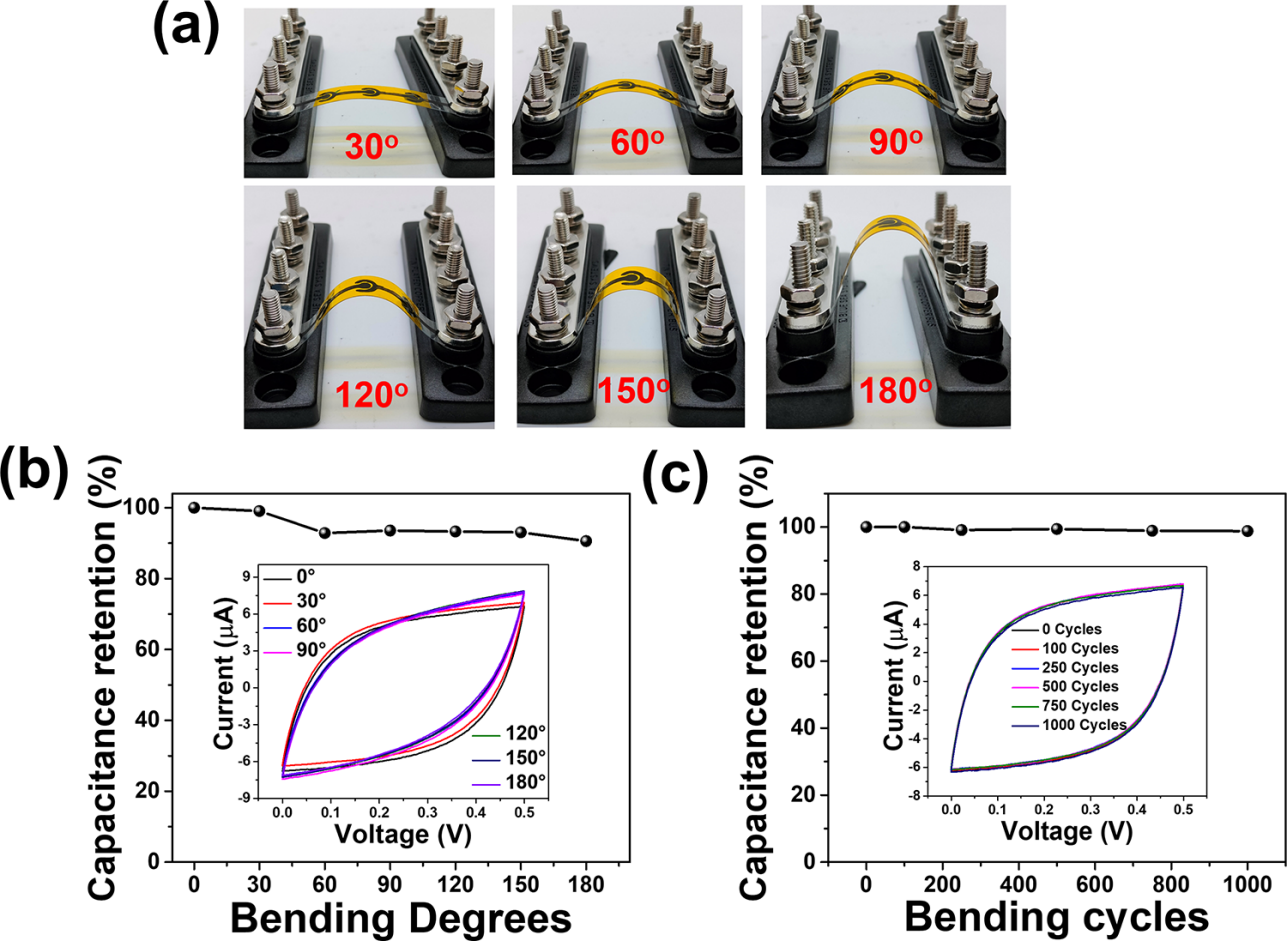
Capacitance retention study (mechanical stability), (a) CNO-based MSCs connected in 3S × 1P are in bend state at different bending angles (30, 60, 90, 120, 150 and 180), (b) calculated capacitance from CV measured at different bending angles (inset shows the CVs), and (c) calculated capacitance from CV measured at different bending cycles (inset shows the CVs).

To demonstrate real-life applications of printed MSCs, a 6S × 3P CNO-IMSC configuration was fabricated and used to power an light emitting diode (LED). The printed device was charged to 3 V by using a Gamry system and then connected to an LED on a breadboard. As shown in Figure 8, the LED illuminated within just 10 s. This setup is also shown in Video S1 in the Supporting Information. This demonstration effectively confirms the practical utility of printed MSCs with CNOs for integration into flexible microelectronics.

**Figure 8:**
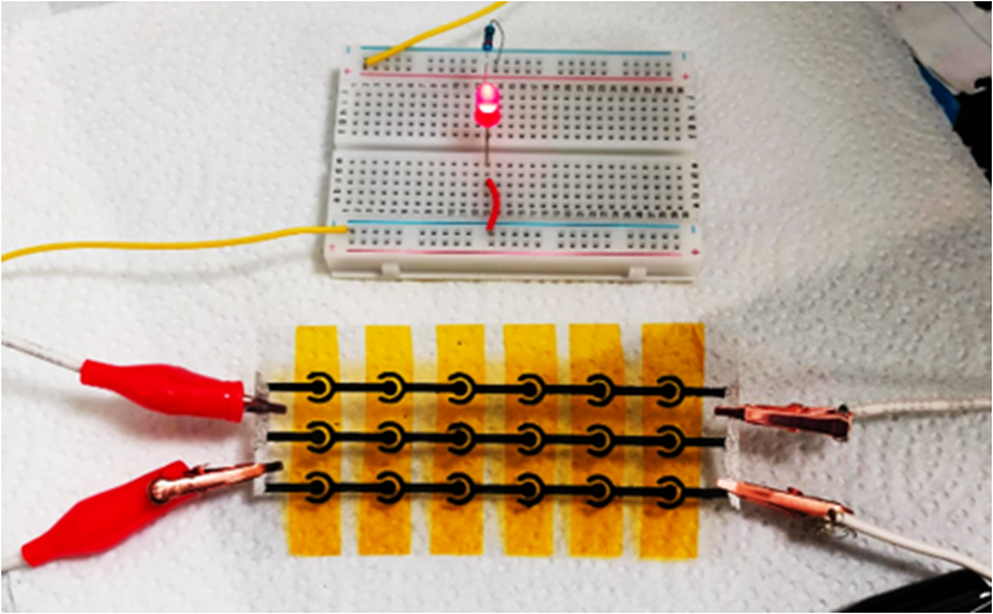
LED lights up using printed MSCs with CNOs connected 6 in series and 3 in parallel.

## 4 Conclusions

This study highlights the potential of hydrocarbon-derived carbon nano-onions in advancing flexible printed electronics, particularly microsupercapacitors (MSCs). By leveraging the unique networked structure of CNOs, these printed MSCs demonstrate a high specific capacitance, superior cycling stability, and excellent capacitance retention, even under extreme bending conditions. By eliminating the need for metal current collectors, CNO-based electrodes provide a lightweight, cost-effective solution, marking a substantial advancement over traditional materials. The performance comparison of CNO-MSCs with existing literature on carbon nanomaterial-based MSCs shows comparable performance, indicating the potential of CNOs as an electrode material for printed MSCs.

## Supplementary Material





## Notes

A video is shown of a printed device charging to 3V by using a Gamry system and then connected to an LED on a breadboard; the LED is illuminated within just 10 s (MP4)
Additional HR-TEM images, detailed material characterization of pristine CNOs (XRD, Raman, and XPS), Raman analysis of printed CNO electrodes, a scan rate vs current plot for *b*-value calculation, conductivity measurements of printed CNO electrodes, Coulombic efficiency plots for CNO-based MSCs, as well as a comprehensive assessment of the morphological, structural, and chemical stability of CNOs after 10,000 GCD cycles; additionally, a performance comparison of CNO-based MSCs with previously reported literature is presented (PDF)
