# Supplementary material for: Hydrocarbon-Derived Prenetworked Carbon Nano-Onions for Wearable and Flexible Printed Microsupercapacitors
Source: ACS Appl Mater Interfaces. 2025 May 27;17(23):34494–503. doi: 10.1021/acsami.5c02847 (PMC12163921; doi:10.1021/acsami.5c02847)
Supplement: Supplementary file 2 [file am5c02847_si_002.pdf]

Supplementary information:

Hydrocarbon-derived pre-networked carbon nano-onions for wearable and flexible printed micro-supercapacitors

Ramu Banavath[1], Yufan Zhang[1], Sayyam Deshpande[1], Smita Shivraj Dasari[1], Stephnie Peat[3], Joseph V. Kosmoski[3], Evan C. Johnson[3], Micah J. Green[1,2 *]

1 Artie McFerrin Department of Chemical Engineering, Texas A&M University, College Station, Texas, 77843 USA

2 Department of Material Science and Engineering, Texas A&M University, College Station, Texas, 77843 USA

3 Nabors Energy Transition Solutions LLC, Houston, Texas, 77067 USA

*Corresponding author: micah.green@tamu.edu

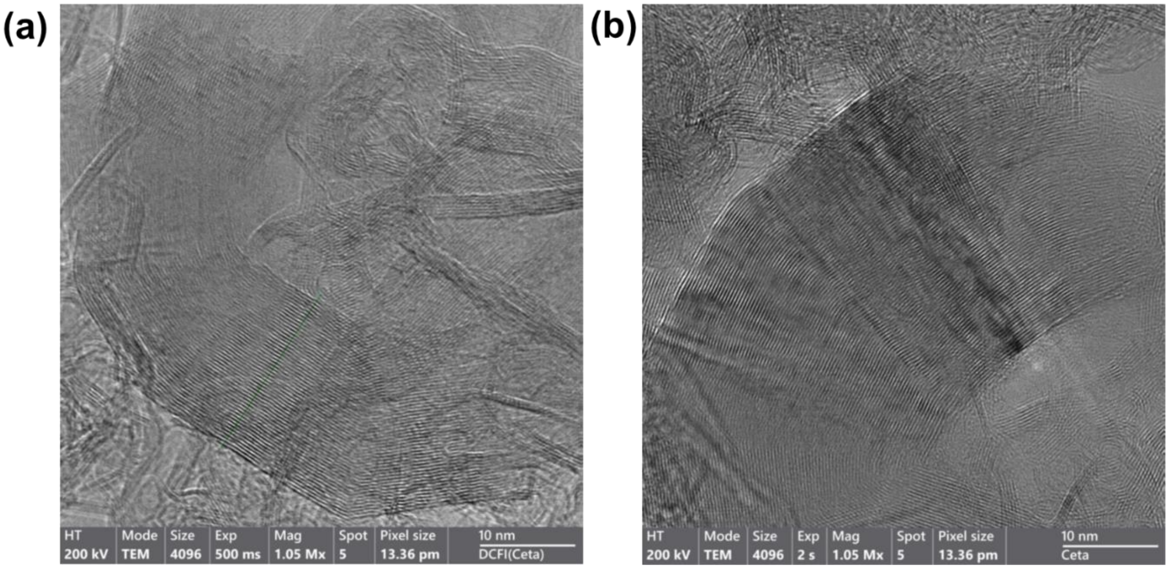

Figure S1: High-resolution TEM images of the pre-networked CNO particles (a) particle 1 and (b) particle 2.

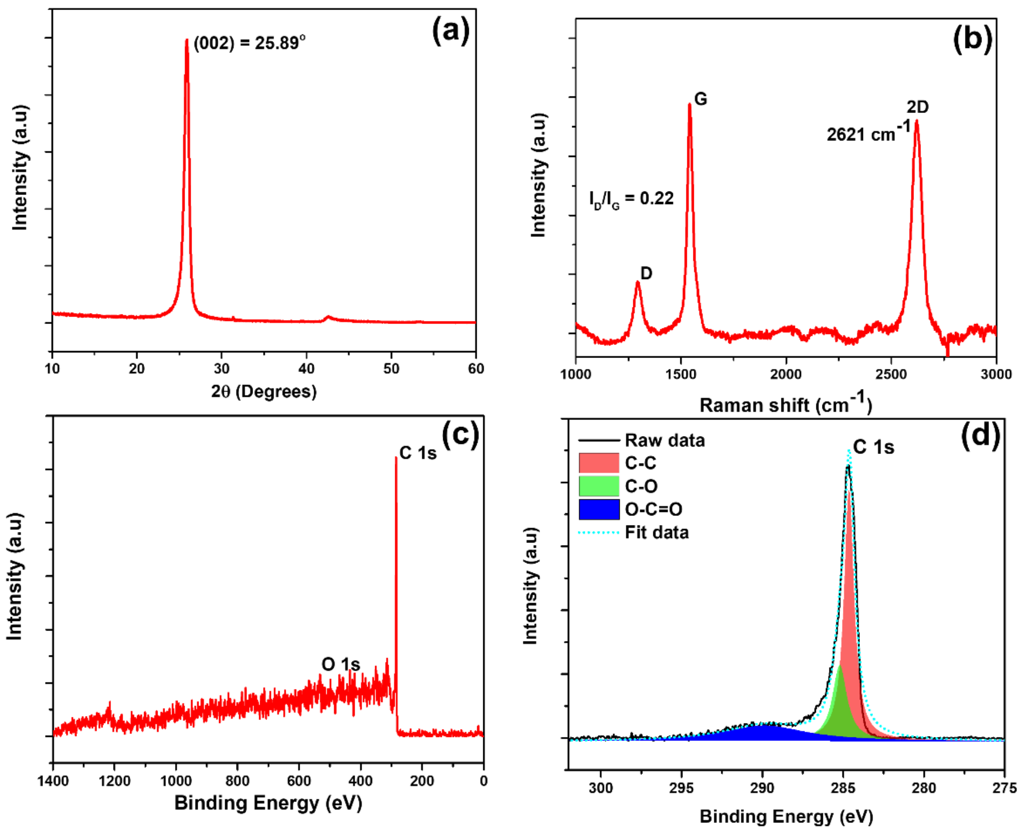

Figure S2: Material characterization of hydrocarbon-derived graphene nanoparticles (GNPs) (a) X-ray diffractometry, (b) Raman spectra of GNPs, (c) XPS survey spectrum of GNPs, and (d) C1s high-resolution XRS spectra of GNPs.

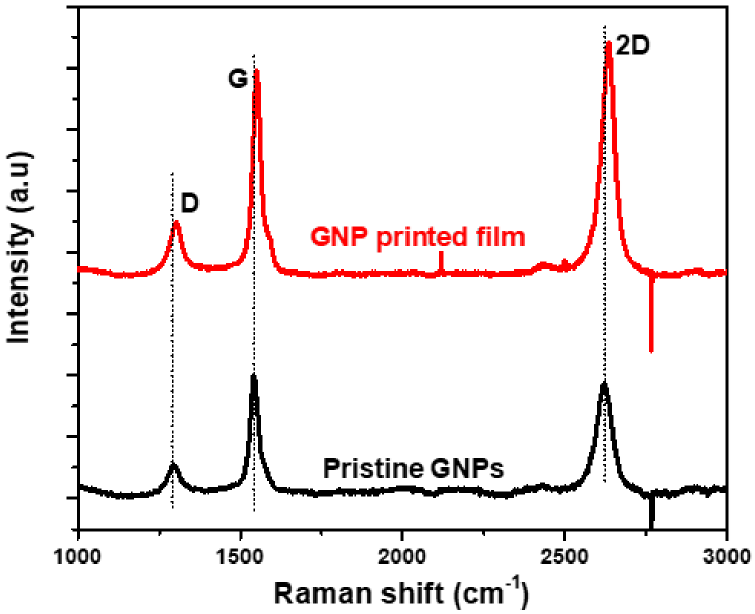

Figure S3: Raman spectra of GNPs and GNP-based printed film.

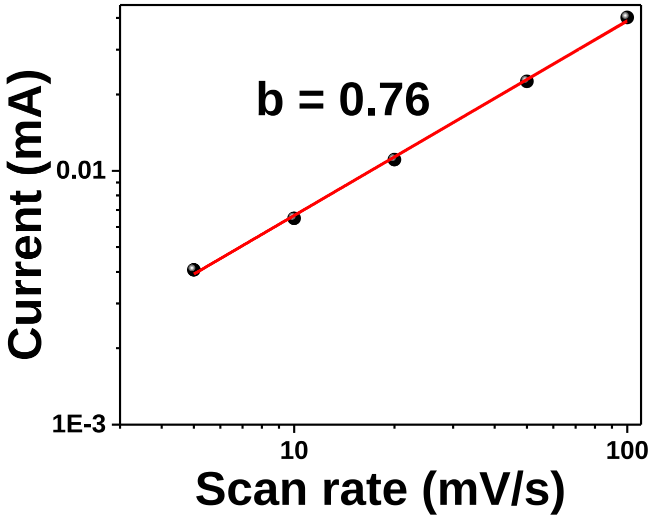

*Figure S4: Current plot vs. scan rate for b-value calculation with equation: log(current(mA)) = log (a) + b*log(scan rate (mV/s)).*

Table S1: The electrical conductivity of CNO, CNT, and Super P-printed films prepared with the same composition.

**Sample**	**Conductivity (S/cm)**
CNO	0.716
CNT	1.921
Super P	1.381

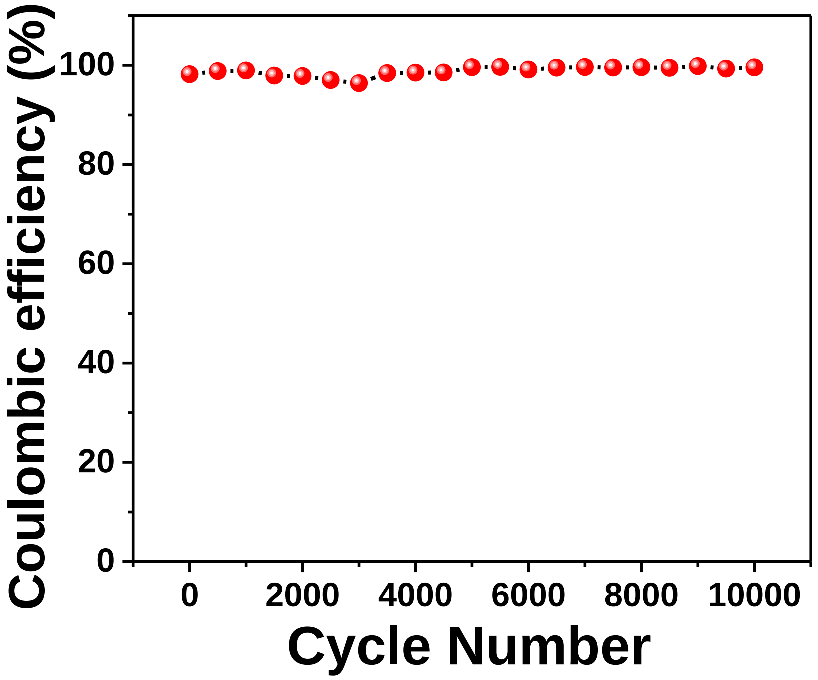

Figure S5: Coulombic efficiency of GNP-based MSC from 0 to 10,000 GCD cycles

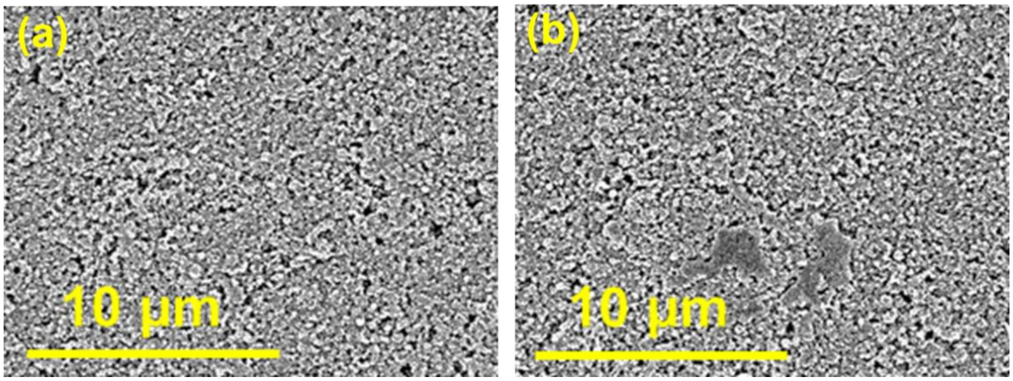

Figure S6: SEM images of CNO electrodes (a) before and (b) after 10,000 GCD cycles.

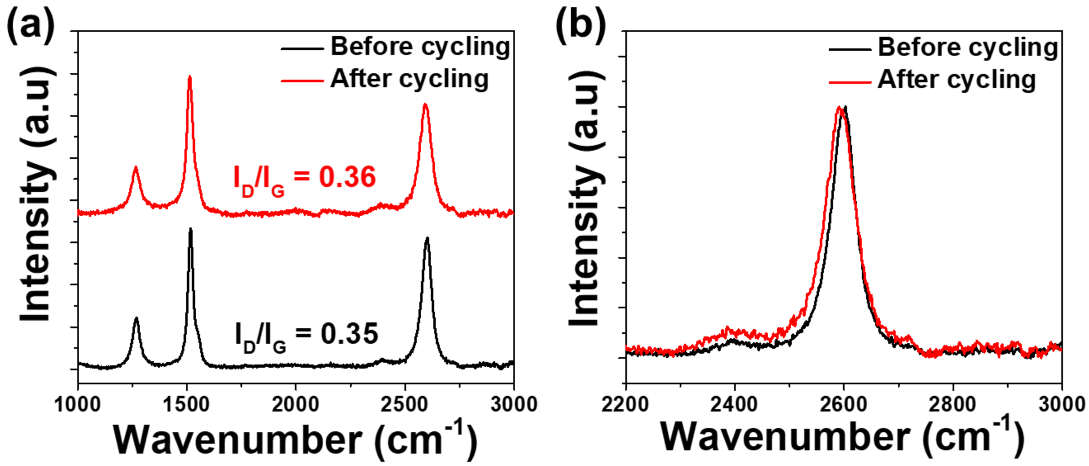

Figure S7: (a) Raman spectroscopy of printed films with CNOs before and after 10,000 GCD cycles, and (b) 2D band of Raman spectroscopy of printed films with CNOs before and after 10,000 GCD cycles.

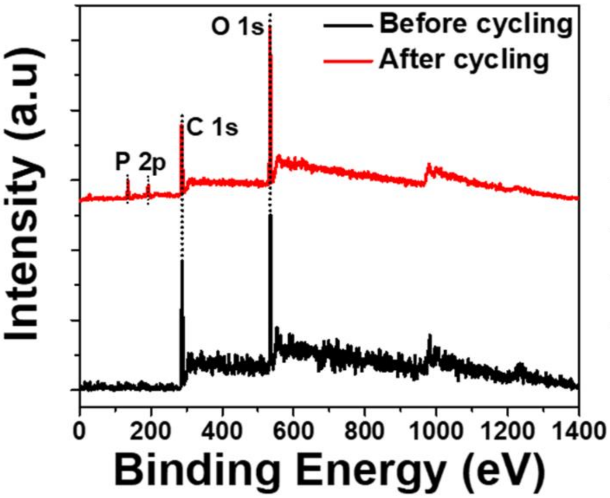

Element	Before cycling	After cycling
C 1s (wt.%)	75.07	57.78
O 1s (wt.%)	24.93	33.03
P 2p (wt.%)	-	9.20

Figure S8: XPS of MSCs before and after 10,000 GCD cycles.

Table S2: The performance comparison of CNO-based MSCs with literature.

Material	Printing Technique	Specific Capacitance ($mF\ cm^{-2}$)	Reference
CNO-MSCs	Screen-Printing	3.8 at 5 mV/s	This work
Graphene MSCs	Screen-Printing	1.0 at 5 mV/s	[1]
Graphene MSCs	Inkjet Printing	0.7 at 10 mV/s	[2]
Graphene MSCs	Flash foam stamping method	4.02 at 10 mV/s	[3]
Graphene MSCs	Chemical Vapor Deposition	1.06 at 0.1 mA cm^{-2}	[4]
GP-MSCs	Spray Coating	4.9 at 2 mV/s	[5]
RuO_2/AgNWs/GO	Screen-Printing	26 at 1 mV/s	[6]
MoS_2/EEG MSCs	Screen-Printing	8.2 at 5 mV/s	[7]
Graphene MSCs	Spray Coating	6.6 at 1 mV/s	[8]
Graphene MSCs	Vacuum filtration	9.5 at 5 mV/s	[9]

Video S1: Video corresponding to Figure 8, real-time charging of fabricated supercapacitors

References:

(1) Shi, X.; Pei, S.; Zhou, F.; Ren, W.; Cheng, H.-M.; Wu, Z.-S.; Bao, X. Ultrahigh-voltage integrated micro-supercapacitors with designable shapes and superior flexibility. *Energy & Environmental Science* **2019**, *12* (5), 1534-1541, 10.1039/C8EE02924E. DOI: 10.1039/C8EE02924E.

(2) Li, J.; Sollami Delekta, S.; Zhang, P.; Yang, S.; Lohe, M. R.; Zhuang, X.; Feng, X.; Östling, M. Scalable Fabrication and Integration of Graphene Microsupercapacitors through Full Inkjet Printing. *ACS Nano* **2017**, *11* (8), 8249-8256. DOI: 10.1021/acsnano.7b03354.

(3) Zhao, J.; Shi, Q.; Guo, Y.; Wang, X.; Wang, D.; Tan, F.; Jiang, L.; Yu, Y. Flash foam stamp-inspired fabrication of flexible in-plane graphene integrated micro-supercapacitors on paper. *Journal of Power Sources* **2019**, *433*, 226703. DOI: https://doi.org/10.1016/j.jpowsour.2019.226703.

(4) Yao, Z.; Quan, B.; Yang, T.; Li, J.; Gu, C. Flexible supercapacitors based on vertical graphene/carbon fabric with high rate performance. *Applied Surface Science* **2023**, *610*, 155535. DOI: https://doi.org/10.1016/j.apsusc.2022.155535.

(5) Shi, X.; Wu, Z.-S.; Qin, J.; Zheng, S.; Wang, S.; Zhou, F.; Sun, C.; Bao, X. Graphene-Based Linear Tandem Micro-Supercapacitors with Metal-Free Current Collectors and High-Voltage Output. *Advanced Materials* **2017**, *29* (44), 1703034. DOI: https://doi.org/10.1002/adma.201703034.

(6) Li, H.; Liu, S.; Li, X.; Wu, Z.-S.; Liang, J. Screen-printing fabrication of high volumetric energy density micro-supercapacitors based on high-resolution thixotropic-ternary hybrid interdigital micro-electrodes. *Materials Chemistry Frontiers* **2019**, *3* (4), 626-635, 10.1039/C8QM00639C. DOI: 10.1039/C8QM00639C.

(7) Yang, W.; Hu, Z.; Zhang, C.; Guo, Y.; Zhao, J. Screen printing preparation of high-performance flexible planar micro-supercapacitors based on MoS2 nanoparticles decorated electrochemically exfoliated graphene. *Electrochimica Acta* **2022**, *429*, 141041. DOI: https://doi.org/10.1016/j.electacta.2022.141041.

(8) Zheng, S.; Tang, X.; Wu, Z.-S.; Tan, Y.-Z.; Wang, S.; Sun, C.; Cheng, H.-M.; Bao, X. Arbitrary-Shaped Graphene-Based Planar Sandwich Supercapacitors on One Substrate with Enhanced Flexibility and Integration. *ACS Nano* **2017**, *11* (2), 2171-2179. DOI: 10.1021/acsnano.6b08435.

(9) Xiao, H.; Wu, Z.-S.; Chen, L.; Zhou, F.; Zheng, S.; Ren, W.; Cheng, H.-M.; Bao, X. One-Step Device Fabrication of Phosphorene and Graphene Interdigital Micro-Supercapacitors with High Energy Density. *ACS Nano* **2017**, *11* (7), 7284-7292. DOI: 10.1021/acsnano.7b03288.
